# Prevention paradox: Medical students are less inclined to prescribe HIV pre‐exposure prophylaxis for patients in highest need

**DOI:** 10.1002/jia2.25147

**Published:** 2018-06-22

**Authors:** Sarah K Calabrese, Valerie A Earnshaw, Kristen Underhill, Douglas S Krakower, Manya Magnus, Nathan B Hansen, Kenneth H Mayer, Joseph R Betancourt, Trace S Kershaw, John F Dovidio

**Affiliations:** ^1^ Department of Psychology George Washington University Washington DC USA; ^2^ Social and Behavioral Sciences Department Yale School of Public Health Yale University New Haven CT USA; ^3^ Department of Human Development and Family Studies University of Delaware Newark DE USA; ^4^ Columbia Law School Columbia University New York NY USA; ^5^ Heilbrunn Department of Population & Family Health Mailman School of Public Health Columbia University New York NY USA; ^6^ The Fenway Institute Fenway Health Boston MA USA; ^7^ Beth Israel Deaconess Medical Center Harvard Medical School Harvard University Boston MA USA; ^8^ Department of Population Medicine Harvard Medical School Harvard University Boston MA USA; ^9^ Department of Epidemiology and Biostatistics Milken Institute School of Public Health George Washington University Washington DC USA; ^10^ Department of Health Promotion and Behavior College of Public Health University of Georgia Athens GA USA; ^11^ Department of Global Health and Population Harvard TH Chan School of Public Health Harvard University Boston MA USA; ^12^ Disparities Solutions Center Massachusetts General Hospital Harvard Medical School Harvard University Boston MA USA; ^13^ Department of Psychology Yale University New Haven CT USA

**Keywords:** HIV, pre‐exposure prophylaxis, healthcare disparities, prescriptions, health personnel, sexual minorities

## Abstract

**Introduction:**

Despite healthcare providers’ growing awareness of pre‐exposure prophylaxis (PrEP), prescription rates remain low. PrEP is an efficacious HIV prevention strategy recommended for use with condoms but still protective in their absence. Concern about the impact of PrEP on condom use and other risk behaviour is, nonetheless, among the barriers to prescription commonly reported. To understand the implications of this concern for PrEP access, we examined how medical students’ willingness to prescribe PrEP varied by patients’ condom use and partnering practices. We also assessed the perceived acceptability of various reasons for condom discontinuation with PrEP.

**Methods:**

An online survey was distributed to 854 medical students in the Northeastern US in 2015. Participants (n = 111) were surveyed about their willingness to prescribe PrEP for each of six male patients who systematically differed in their reported condom use (sustained use, sustained nonuse, or discontinuation with PrEP) and partnering practices (single male partner with untreated HIV or multiple male partners of unknown HIV status). Participants also reported perceived acceptability of four reasons for condom discontinuation: pleasure, sexual functioning, intimacy, and conception.

**Results:**

Willingness to prescribe PrEP was inconsistent with patient risk: When the patient used condoms and planned to sustain condom use, most participants were willing to prescribe PrEP – 93% if the patient had a single partner and 86% if the patient had multiple partners. Fewer were willing to prescribe if the patient did not use condoms and planned to sustain nonuse (53% and 45%, respectively) or used condoms but planned to discontinue use (27% and 28%). Significantly fewer participants were willing to prescribe for a patient with multiple partners versus a single partner when the patient reported sustained condom use or sustained condom nonuse. The number of participants who were willing to prescribe was similarly low for a patient with multiple partners versus a single partner when the patient reported that he planned to discontinue condom use. More participants accepted a patient discontinuing condoms for conception (69%) than for intimacy (23%), pleasure (14%), or sexual functioning (13%).

**Conclusion:**

Medical students’ clinical judgments were misaligned with patient risk and suggest misconceptions or personal values may undermine provision of optimal HIV prevention services.

## 1 Introduction

Pre‐exposure prophylaxis (PrEP) is an efficacious HIV prevention strategy 1. US clinical guidelines cite condomless sex as a key indicator of HIV risk and PrEP candidacy 2. However, many healthcare providers have expressed concern that condomless sex and other risk behaviours will increase if patients are prescribed PrEP 3, 4, 5, 6, 7, 8, 9, 10, 11, 12, 13, 14, 15, 16. This sets up a potential “prevention paradox:” Some providers may be more willing to prescribe PrEP for patients who would use condoms concurrently with PrEP than for patients who would not, even though the latter would be in greater need of PrEP absent other forms of protection. In the current survey study of medical students, we examined participants’ willingness to prescribe PrEP for patients whose condom use and partnering practices systematically differed. We also explored the perceived acceptability of various reasons for patients discontinuing condom use while taking PrEP.

### 1.1 Background

The updated US clinical guidelines 2 include recommendations to consider PrEP for sexually active, HIV‐uninfected men who have sex with men (MSM) who are not in a monogamous relationship with a recently tested, HIV‐negative man and have engaged in recent condomless anal sex or recently had a bacterial sexually transmitted infection (STI). The guidelines also offer specific PrEP indications for people at risk for HIV because of heterosexual activity or injection practices.

Despite the availability of clinical guidelines to support its implementation, many providers have not prescribed PrEP. A 2015 national survey of over 1500 US primary care providers found that only 7% had ever prescribed PrEP, even though 66% were aware of it 17. One potential barrier to prescribing PrEP that providers have repeatedly reported is concern about sexual risk compensation, which refers to patients increasing their risk behaviour because of a perceived decrease in HIV susceptibility while taking PrEP 3, 4, 5, 6, 7, 8, 9, 10, 11, 12, 13, 14, 15, 16. Although risk compensation behaviour was not commonly reported by PrEP recipients in early research studies 1, a number of PrEP users seen in clinical or community‐based practice settings have reported reducing their condom use 18, 19 or increasing the number of sexual partners with whom they engage in condomless sex 20 after initiating PrEP. PrEP candidates have identified diverse motivations for reducing condom use while taking PrEP, including conception, intimacy, and sexual pleasure 21, 22, 23.

Importantly, patients’ intentions and motivations surrounding condomless sex and other forms of risk compensation behaviour are not clinically supported reasons for providers to withhold PrEP from patients 24. Although PrEP in combination with condoms might maximize sexual health protection by preventing HIV, other STIs, and unwanted pregnancy, PrEP is still protective against HIV in the absence of condoms. Current scientific evidence suggests that the level of protection that PrEP offers may outweigh the risk incurred by reductions in condom use. For example, across two clinical settings, even though 34% to 41% of the subset of PrEP patients surveyed six to seven months after initiating PrEP reported reducing their condom use, there were no seroconversions among the combined sample of over 1500 patients 18, 19. Modelling studies have corroborated this finding, suggesting that MSM who are fully adherent to PrEP would maintain or increase protection even if they reduced or discontinued condom use 25, 26. Thus, medical evidence should lead providers to be no less willing to prescribe PrEP for patients who engage in condomless sex (or plan to do so) than patients already protected through condom use.

However, factors outside of medical evidence influence providers’ clinical decisions, including cognitive biases related to patient characteristics 27. Patients with identical presenting problems or clinical requests can encounter differential treatment based on seemingly irrelevant social and behavioural characteristics. For example, providers have reported greater willingness to prescribe opioids for pain relief when the patient was injured during a ladder fall versus running from the police 28. To the extent that socially acceptable versus socially stigmatized patient characteristics yield more favourable treatment, providers may be more willing to prescribe PrEP for patients reporting continued condom use and monogamy versus condomless sex and non‐monogamy. This discrepant treatment would undermine access for those most in need, producing a prevention paradox.

### 1.2 Study overview and objectives

In this survey study of medical students, we systematically examined how PrEP clinical decision‐making varied based on patients’ reported sexual behaviour and motivations. First, we assessed the effects of patient condom use (sustained use versus sustained nonuse versus planned discontinuation with PrEP), patient partnering practices (single male partner with untreated HIV versus multiple male partners of unknown HIV status), and their interaction on participants’ willingness to prescribe PrEP. Specifically, participants rated their willingness to prescribe PrEP for six HIV‐negative male patients reporting different combinations of condom use and partnering practices. If participants’ judgment aligned with medical evidence, willingness to prescribe PrEP would be expected to correspond to patient risk and be high for all six patients (because all reported high‐risk behaviour) and highest for patients reporting condomless sex.

Second, we assessed the perceived acceptability of a patient discontinuing condoms while taking PrEP when motivated by conception versus other reasons – pleasure, sexual functioning, and intimacy. If participants’ judgment aligned with medical evidence, similar levels of acceptability would be expected across reasons for condom discontinuation because discontinuation confers the same level of risk irrespective of reason.

## 2 Methods

This survey was conducted as part of a larger study. Procedures described below have been reported elsewhere 29.

### 2.1 Participants and procedures

In 2015, an online survey was distributed through internal email lists to 854 medical students attending two medical schools in the northeastern US. After consenting to participate, they were presented with background information about PrEP (e.g. clinical efficacy) and supporting and opposing claims for prescribing PrEP 30; completed survey measures; and received compensation. (See Data S1 for survey background information, claims, and primary measures). The Yale University Human Subjects Committee approved all procedures.

### 2.2 Measures

#### 2.2.1 Willingness to prescribe PrEP

Participants rated their likelihood of prescribing PrEP to six HIV‐uninfected male patients. Patients’ reported behaviour varied systematically according to a 3 (patient condom use) × 2 (patient partnering practices) within‐subjects design, such that a different combination of condom use and partnering practices was described for each patient. The three condom use categories included *sustained condom use* (uses condoms and wants to continue using condoms with PrEP), *sustained nonuse* (does not use condoms and wants to continue not using condoms with PrEP), and *planned discontinuation* (uses condoms and wants to stop using condoms with PrEP). The two partnering practice categories were *single partner* (in a monogamous relationship with a man who has HIV and is not on treatment) and *multiple partners* (has sex with multiple men whose HIV statuses he does not know). Responses were recoded to create a dichotomous variable: willing (“very” or “extremely” likely) versus not willing (“not at all,” “a little bit,” or “somewhat” likely) to prescribe.

#### 2.2.2 Perceived acceptability of reasons for condom discontinuation

Perceived acceptability of reasons for condom discontinuation was assessed with one question: “Which of the following reasons (if any) are acceptable reasons for a male patient to stop using condoms while on PrEP? (check all that apply).” The four response options were “because he finds sex without condoms to be more physically pleasurable,” “because it is easier for him to maintain an erection (stay hard) without condoms,” “because he feels closer and more emotionally connected to his partner without condoms,” and “because he is trying to conceive (get pregnant) with his female partner.” These reasons for discontinuation represented *pleasure*,* sexual functioning*,* intimacy*, and *conception*, respectively.

#### 2.2.3 Background characteristics

Participants reported sociodemographic information, including age, race/ethnicity, gender, and sexual orientation. They also reported their familiarity with PrEP (recoded as ever versus never heard of PrEP), prior PrEP education (ever versus never learned about PrEP as part of their medical school training), and years of medical school completed.

### 2.3 Analysis

Frequency distributions were calculated to characterize the sample. Logistic regressions using generalized estimating equations (because of the within‐subjects design) were performed to examine additive and multiplicative (i.e. interactive or moderated) effects of patient condom use and partnering practices on willingness to prescribe PrEP, and to explore differences in the perceived acceptability of condom discontinuation with PrEP across four reasons. Analyses were repeated adjusting for relevant background characteristics. We adjusted for age, race/ethnicity, and prior PrEP education because these characteristics were statistically related to one or both outcomes. We adjusted for gender and sexual orientation given their conceptual relevance.

## 3 Results

### 3.1 Sample characteristics

Of the 854 medical students contacted, 169 enrolled in the study and 111 completed all relevant measures, yielding a 13% response rate. As compared to the combined enrollment statistics for the two medical schools, a larger percentage of our study sample was White (62% of study sample versus 50% of all medical students; χ^2^ [1] = 6.57, *p* = 0.01) and female (66% versus 49%, respectively; χ^2^ [1] = 13.02, *p* < 0.01). Table 1 displays additional sample characteristics.

**Table 1:** Sample characteristics (n = 111)

	n (%)
Age	
<25 years	62 (55.9)
≥25 years	49 (44.1)
Race/ethnicity	
White	69 (62.2)
Asian	30 (27.0)
Black/African American	7 (6.3)
Latino/Hispanic	3 (2.7)
Other	2 (1.8)
Gender	
Female	73 (65.8)
Male	37 (33.3)
Other	1 (0.9)
Sexual orientation	
Heterosexual	96 (86.5)
Bisexual	6 (5.4)
Gay/Lesbian	6 (5.4)
Other	3 (2.7)
Years of medical school completed	
<1 (currently in first year)	24 (21.6)
1 (currently in second year)	37 (33.3)
2 (currently in third year)	27 (24.3)
≥3 (currently in fourth year+)	23 (20.7)
PrEP familiarity	
Heard of PrEP	94 (84.7)
Never heard of PrEP	17 (15.3)
Prior PrEP education	
Learned about PrEP in medical school	56 (50.5)
Did not learn about PrEP in medical school	55 (49.5)

PrEP, pre‐exposure prophylaxis.

### 3.2 Effects of patient condom use and partnering practices on willingness to prescribe PrEP

**Figure 1:**
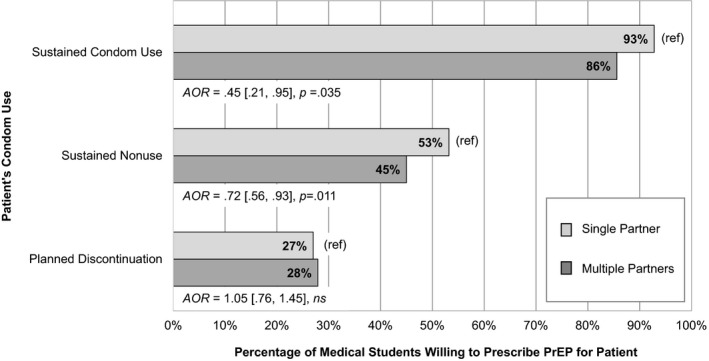
Medical students’ willingness to prescribe pre‐exposure prophylaxis (PrEP) for a hypothetical male patient. Patient condom use and partnering practices were systematically varied using a 3 × 2 within‐subjects design such that every participant rated six patients with differing combinations of condom use and partnering practices. AORs represent the effect of partnering practice on willingness to prescribe within each condom use category, adjusting for relevant background characteristics (age, race/ethnicity, gender, sexual orientation, and prior PrEP education).

Figure 1 displays the percentage of participants who were willing to prescribe PrEP for each of the six hypothetical patients. Tables 2 and 3 present additive and multiplicative effects. In the initial (additive) regression model, condom use and partnering practices were significantly associated with willingness to prescribe PrEP. For the lowest‐risk condom use category (sustained condom use), most participants were willing to prescribe – 86% if the patient was described as having multiple male partners and 93% if the patient had a single partner. Fewer were willing to prescribe if the patient did not use condoms and planned to sustain nonuse (45% if multiple partners, 53% if single partner) or used condoms but planned to discontinue use (28% if multiple partners, 27% if single partner). Overall, fewer participants were willing to prescribe if the patient had multiple male partners versus a single partner.

**Table 2:** Additive and interaction effects of condom use and partnering practices on willingness to prescribe PrEP

Model	Unadjusted			Adjusted^a^		
	Wald χ^2^	df	*p*	Wald χ^2^	df	*p*
1. Additive effects						
Condom use	100.42	2	<0.001	100.76	2	<0.001
Partnering practice	5.79	1	0.016	6.21	1	0.013
2. Conditional and interaction effects						
Condom use	95.73	2	<0.001	96.40	2	<0.001
Partnering practice	5.67	1	0.017	5.85	1	0.016
Condom use × partnering practice	6.63	2	0.036	6.57	2	0.037

PrEP, pre‐exposure prophylaxis.

a Model adjusted for relevant background characteristics (age, race/ethnicity, gender, sexual orientation, and prior PrEP education).

**Table 3:** Comparisons of willingness to prescribe PrEP across condom use categories and partnering practices (additive model)

	Unadjusted			Adjusted^a^		
	OR	95% CI	*p*	AOR	95% CI	*p*
Sustained condom use (ref)	‐	‐	‐	‐	‐	‐
Sustained nonuse	0.14	0.08, 0.23	<0.001	0.14	0.09, 0.23	<0.001
Planned discontinuation	0.05	0.03, 0.09	<0.001	0.05	0.03, 0.09	<0.001
Single partner (ref)	‐	‐	‐	‐	‐	‐
Multiple partners	0.77	0.63, 0.95	0.016	0.76	0.61, 0.94	0.013

PrEP, pre‐exposure prophylaxis.

a Model adjusted for relevant background characteristics (age, race/ethnicity, gender, sexual orientation, and prior PrEP education).

When the condom use x partnering practice interaction term was added to the model, a significant interaction was detected. As shown in Table 4, follow‐up logistic regression analyses examining partnering practice effects stratified by condom use revealed that fewer participants were willing to prescribe if the patient had multiple partners versus a single partner for two of three condom use categories: sustained condom use and sustained condom nonuse.

**Table 4:** Comparison of willingness to prescribe PrEP for patients with single versus multiple partners, stratified by condom use

Condom use category	Partnering practice	Unadjusted			Adjusted^a^		
		OR	95% CI	*p*	AOR	95% CI	*p*
Sustained condom use	Single partner (ref)	‐	‐	‐	‐	‐	‐
	Multiple partners	0.46	0.23, 0.94	0.033	0.45	0.21, 0.95	0.035
Sustained nonuse	Single partner (ref)	‐	‐	‐	‐	‐	‐
	Multiple partners	0.72	0.56, 0.93	0.011	0.72	0.56, 0.93	0.011
Planned discontinuation	Single partner (ref)	‐	‐	‐	‐	‐	‐
	Multiple partners	1.05	0.76, 1.44	0.781	1.05	0.76, 1.45	0.782

PrEP, pre‐exposure prophylaxis.

To probe the interaction, the effect of partnering practice was examined in separate models for sustained condom use, sustained nonuse, and planned discontinuation.

a Model adjusted for relevant background characteristics (age, race/ethnicity, gender, sexual orientation, and prior PrEP education).

### 3.3 Differences in perceived acceptability of reasons for condom discontinuation

Perceived acceptability of discontinuation varied across reasons. Most participants (69%) considered conception an acceptable reason for condom discontinuation, but fewer accepted intimacy (23%), pleasure (14%), or sexual functioning (13%; see Table 5).

**Table 5:** Perceived acceptability of reasons a man may discontinue condoms while taking PrEP

Reason for discontinuing condoms	Participants reporting reason to be acceptable^a^	Unadjusted				Adjusted^b^	
	n (%)	OR	95% CI	*p*	AOR	95% CI	*p*
Conception (ref)	76 (68.5)	‐	‐	‐	‐	‐	‐
Intimacy	26 (23.4)	0.14	0.08, 0.24	<0.001	0.14	0.08, 0.24	<0.001
Pleasure	16 (14.4)	0.08	0.04, 0.15	<0.001	0.08	0.04, 0.14	<0.001
Sexual functioning	14 (12.6)	0.07	0.04, 0.13	<0.001	0.07	0.03, 0.13	<0.001

PrEP, pre‐exposure prophylaxis.

a Reasons were not mutually exclusive (participants could report multiple reasons to be acceptable).

b Model adjusted for relevant background characteristics (age, race/ethnicity, gender, sexual orientation, and prior PrEP education).

## 4 Discussion

Medical students’ judgments were fundamentally misaligned with medical evidence and suggested a prevention paradox, whereby patients more in need of PrEP were less likely to receive it. First, whereas the vast majority of participants were willing to prescribe PrEP for a condom‐using patient who planned to keep using condoms while taking PrEP (lowest risk condom use category), far fewer were willing to prescribe PrEP for a patient who planned to stop using condoms or did not use condoms to begin with. Second, although the partnering practices of all patients suggested high risk, more participants were willing to prescribe when the patient was described as having a single partner versus multiple partners in most circumstances. Finally, even though condom discontinuation confers the same level of risk regardless of underlying motivation, more participants were accepting of condom discontinuation for the purpose of conception than for intimacy, pleasure, or sexual functioning. Collectively, these findings suggest that PrEP‐related clinical judgments may be vulnerable to misconceptions – about PrEP, risk compensation, and/or standards of clinical practice – as well as other decisional influences, such as personal values surrounding condom use, monogamy, and other aspects of sexuality.

The variation in participants’ willingness to prescribe PrEP across condom categories is inconsistent with medical evidence and only partially consistent with risk compensation concerns. That fewer participants were willing to prescribe PrEP for condom users planning to discontinue versus sustain condom use may indeed reflect concern that condom discontinuation with PrEP could increase HIV and other STI risk. However, risk compensation concerns do not explain why fewer participants were willing to prescribe PrEP for patients already forgoing condoms. For most patients who do not use condoms and plan to sustain nonuse – a category that many actual PrEP users fall into 31 – HIV risk would decrease and STI risk would be unlikely to change significantly if prescribed PrEP. Greater reluctance to prescribe for condom nonusers suggests that misconceptions about the effectiveness of PrEP as a singular form of protection or values related to condoms may have affected participants’ judgment. Notably, this finding is inconsistent with prior studies of HIV care providers reporting condom nonuse to be an indicator of PrEP candidacy 32 and reporting greater willingness to prescribe for MSM who “sometimes” versus “always” use condoms 3, which may reflect differences in training and experience.

The variation in participants’ willingness to prescribe PrEP across partnering practices, whereby more participants were willing to prescribe for patients with a single partner versus multiple partners in most circumstances, suggests values related to monogamy may have clouded participants’ judgment. Alternatively, participants may have reasoned that patients in an ongoing sexual relationship with a partner with untreated HIV were at higher risk because these patients would potentially be exposed to HIV with every sexual event, whereas patients with multiple partners of unknown HIV status would only be exposed during some sexual events (since some partners would likely be HIV‐negative). The low number of participants willing to prescribe PrEP for MSM planning to discontinue condom use – whether they had one or multiple partners – indicates particularly strong resistance to prescribing to condom users who intend to use PrEP as an alternative (rather than supplemental) form of protection, irrespective of partnering practices.

Higher acceptability of conception over all other reasons for condom discontinuation with PrEP implicates personal values related to sexuality and sexual orientation in participants’ judgments. Given the high level of protection PrEP offers 33, a property that participants were informed of prior to completing survey items, the magnitude of added HIV risk associated with condom discontinuation among adherent patients is likely to be negligible, particularly if prior condom use was suboptimal. The vast majority of participants regarded conception as worthy of that risk, whereas only a third or less regarded increased intimacy, pleasure, and sexual functioning as such. This could suggest that participants placed greater value on sex for reproductive purposes than for other reasons. Alternatively, conception could have been perceived as a more temporary period of discontinuation and, therefore, lower risk. Either way, conception was the only reason of the four that would not apply to same‐sex couples. Thus, the primary reasons for discontinuing condoms that MSM are likely to have were unacceptable to most participants. Sexual prejudice has previously surfaced in qualitative research on providers’ attitudes towards PrEP 5, 34, with some providers openly acknowledging greater comfort prescribing PrEP to serodiscordant heterosexual couples trying to conceive as compared to other patients 5.

### 4.1 Implications for clinical training and guidelines

Provider training curricula and clinical guidelines surrounding PrEP could help to prevent the observed inconsistencies between PrEP‐related clinical judgments and current medical evidence from manifesting in clinical practice.

Within training curricula, common misconceptions related to PrEP, risk compensation, and standards of practice should be addressed directly. Providers should be informed of the relative risks associated with PrEP and condoms as singular and dual forms of protection. Without explicit training about the benefits of PrEP as a singular prevention option, providers may rely on previously learned paradigms equating condomless sex with risk. In addition, alerting providers to the vulnerability of clinical judgment to medically unjustified personal beliefs and biases could lead to greater vigilance and preventive action in practice.

Current US federal guidelines cite condomless sex as an indicator of PrEP eligibility and suggest that PrEP is unnecessary if consistent condom use can be achieved 2. However, the guidelines also suggest that PrEP is intended for concurrent use with condoms and that PrEP patients should be counselled accordingly 2. These mixed messages surrounding the relevance of condom use to PrEP candidacy may foster confusion. Express acknowledgement of PrEP's value and acceptability as a singular form of protection and comprehensive recommendations for counselling patients who choose to forgo condoms (or linkage to such recommendations 35) should be included within clinical guidelines.

Both training curricula and clinical guidelines should instruct providers to discuss PrEP with patients irrespective of patients’ reported sexual behaviour 36. Patients are not always able or willing to report their sexual histories in an accurate manner. If patients seeking PrEP believe that self‐disclosing condomless sex or other risk behaviour to providers would deter providers from prescribing PrEP, a belief supported by the current findings, they may be less inclined to disclose. Nondisclosure would diminish providers’ capacity to accurately assess patients’ HIV risk and other health needs and decrease the quality of care and range of services provided. A patient‐centered approach to sexual healthcare, whereby providers support patients in making informed decisions, should be practiced and communicated to patients. As in HIV treatment, allied health professionals such as pharmacists and counselors may play key roles in PrEP education, prescription, and maintenance, which can help to alleviate the added demands placed on providers.

### 4.2 Limitations

This survey was conducted with medical students, over half of whom were in their first two years of medical school. Although no statistically significant differences in willingness to prescribe PrEP or perceived acceptability of condom discontinuation were detected based on years of medical school, limited clinical instruction and experience reduce the generalizability of our findings to the current health workforce. Participants were recruited in the northeastern US; values related to condoms, monogamy, and other dimensions of sexuality could vary geographically. Generalizability is also limited by the small sample size (n = 111), low response rate (13%), and overrepresentation of White and female students relative to the medical school populations sampled. Additionally, there may have been systematic differences (e.g. in PrEP familiarity) between survey completers and non‐completers that we were unable to characterize, further limiting the external validity of our findings.

The PrEP‐related background information and claims presented are also a potential limitation. Following a paradigm used in prior research 30, we presented factual and empirically supported information (e.g. clinical efficacy 37) as well as claims supporting and opposing PrEP prescription. The claims reflected providers’ self‐reported attitudes in prior research 38, 39. Supporting claims encouraged understanding of patients’ decision to use PrEP without condoms, whereas opposing claims introduced concerns about patient adherence and drug resistance. Though intended to raise key considerations in a relevant and balanced way, this preliminary material may or may not represent messaging that providers would ordinarily encounter or issues that would be salient when making clinical decisions.

In our assessment of prescription willingness, the six patients presented to participants were all MSM. The systematic differences in prescription willingness that we observed across condom use categories may not generalize to other groups for whom condomless sex is less taboo (e.g. heterosexuals). Future research could explore how sexual practices intersect with patient sexual orientation and other characteristics to affect prescription willingness.

Prescription willingness was reported based on hypothetical cases. Hypothetical cases allow researchers to systematically manipulate patient characteristics and make cleaner comparisons and stronger causal inferences. However, exploration of provider reactions to patient self‐disclosures in actual clinical settings would provide a fuller picture.

The repeated‐measures design, according to which each participant rated all six patients, likely drew attention to the differences across patients – namely, condom use and partnering practices, and little additional patient information upon which to base prescription decisions was provided. One might argue that this created an artificial emphasis on these patient behaviours. However, the repeated decision‐making is not unlike clinical practice, in which providers consecutively see patients and make judgments, and patient sexual behaviour is likely to be a central consideration when prescribing PrEP regardless of access to additional patient information. The design may also raise concern that participants were able to discern the study's purpose and potentially motivated to respond in a socially desirable way. However, to the extent that evidence‐based practice is socially desirable, this was not the response pattern observed. Additionally, the ordering of hypothetical patient cases and reasons for condom discontinuation was fixed. Study replication randomizing the order of patient cases and discontinuation reasons would strengthen the inferences made here. Use of a between‐groups design or presentation of additional information to obscure the main research focus could also enhance generalizability of findings.

Finally, we did not examine whether participants accurately assessed the relative risk associated with condoms and PrEP when used as singular and dual forms of protection, nor did we directly measure personal values. Qualitative research may be particularly valuable in exploring the misconceptions, values, and other cognitive and affective processes underlying the clinical judgments reported here 27.

## 5 Conclusions

PrEP and condoms in combination may offer the most comprehensive sexual health protection, but both offer protective benefit and neither is infallible. Withholding PrEP due to condom nonuse or non‐monogamy is not medically justifiable and runs counter to patients’ prevention needs. Our findings suggest a need for PrEP training curricula and clinical guidelines to explicitly support PrEP provision to patients who choose to engage in condomless and non‐monogamous sex and ensure providers realize the value of PrEP as a singular form of HIV protection.

## Competing interest

DSK and KHM have conducted research with unrestricted project support from Gilead Sciences and KHM has conducted research with unrestricted project support from Merck and ViiV Healthcare. (Note: None of this funding was used for the current study). SKC, DSK and KHM have received compensation for their efforts in developing and delivering medical education related to PrEP. The authors declare that they have no other conflicts of interest to disclose.

## Authors’ contributions

SKC, MM, NBH, KHM, JRB, TSK and JFD conceptualized and designed the research study/grant. SKC executed the study and led the research analyses. SKC, VAE, KU, DSK, MM, NBH and JFD contributed to the writing of the manuscript. All authors have read and approved the final manuscript.

## Supporting information


**Data S1.** PrEP background information, claims, and primary measures.
