# Supplementary material for: Prevention paradox: Medical students are less inclined to prescribe HIV pre‐exposure prophylaxis for patients in highest need
Source: J Int AIDS Soc. 2018 Jun 22;21(6):e25147. doi: 10.1002/jia2.25147 (PMC6016621; doi:10.1002/jia2.25147)
Supplement: Supplementary file 1 — Data S1. PrEP background information, claims, and primary measures. [file JIA2-21-e25147-s001.docx]

**Online Supporting Information^[[1]](#footnote-1)^**

**PrEP Background Information, Claims, and Primary Measures**

**
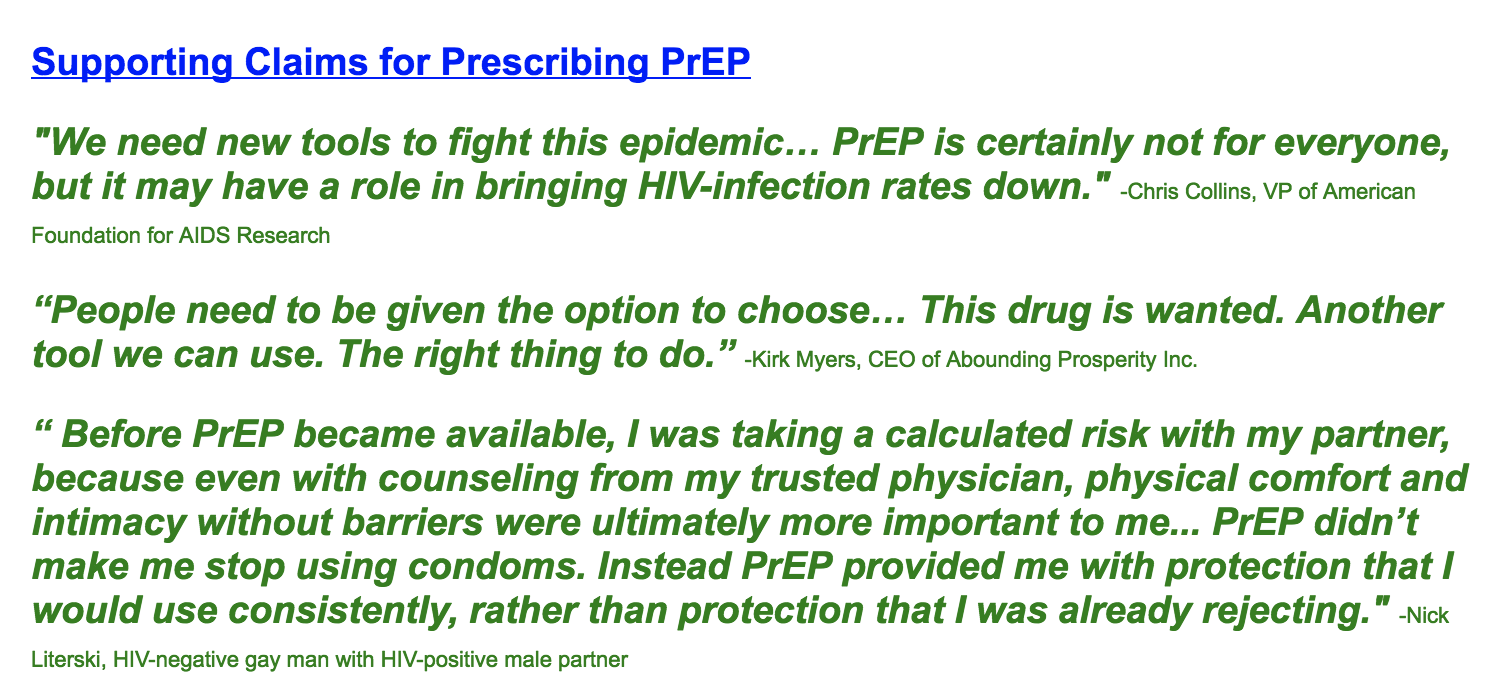

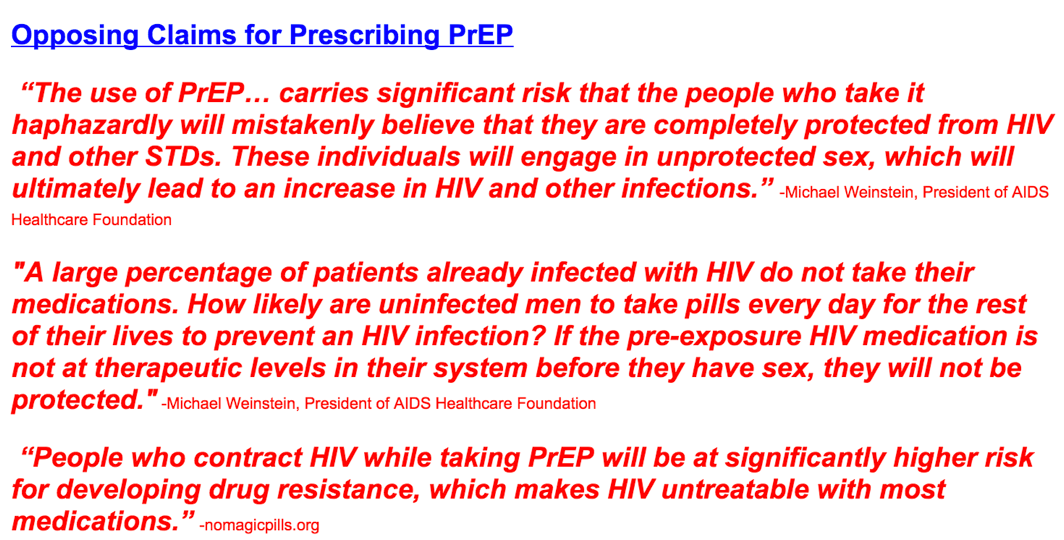
**
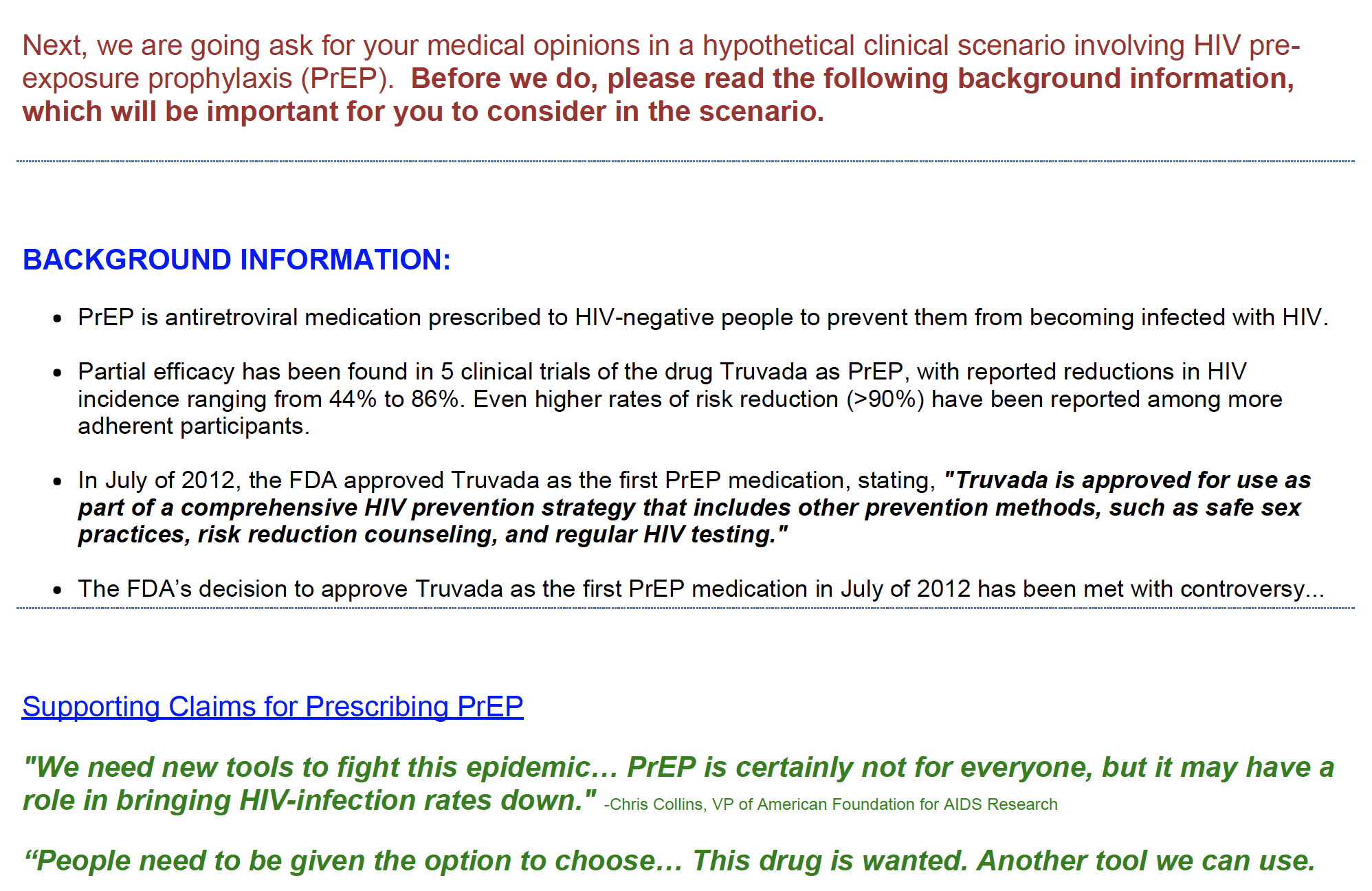
*Background information, claims, and measures are displayed below as formatted in the original Qualtrics survey. Background information and claims were modeled after a prior vignette-based survey study of medical students’ PrEP attitudes [1] with the intention of communicating factual and empirically supported information about PrEP (e.g., US Food and Drug Administration’s approval, clinical efficacy [2]) as well as other key considerations related to prescribing. These other considerations were based on providers’ self-reported attitudes [3,4] and included both substantiated and unsubstantiated concerns at the time the study was conducted.*

*[Willingness to Prescribe PrEP Measure]*


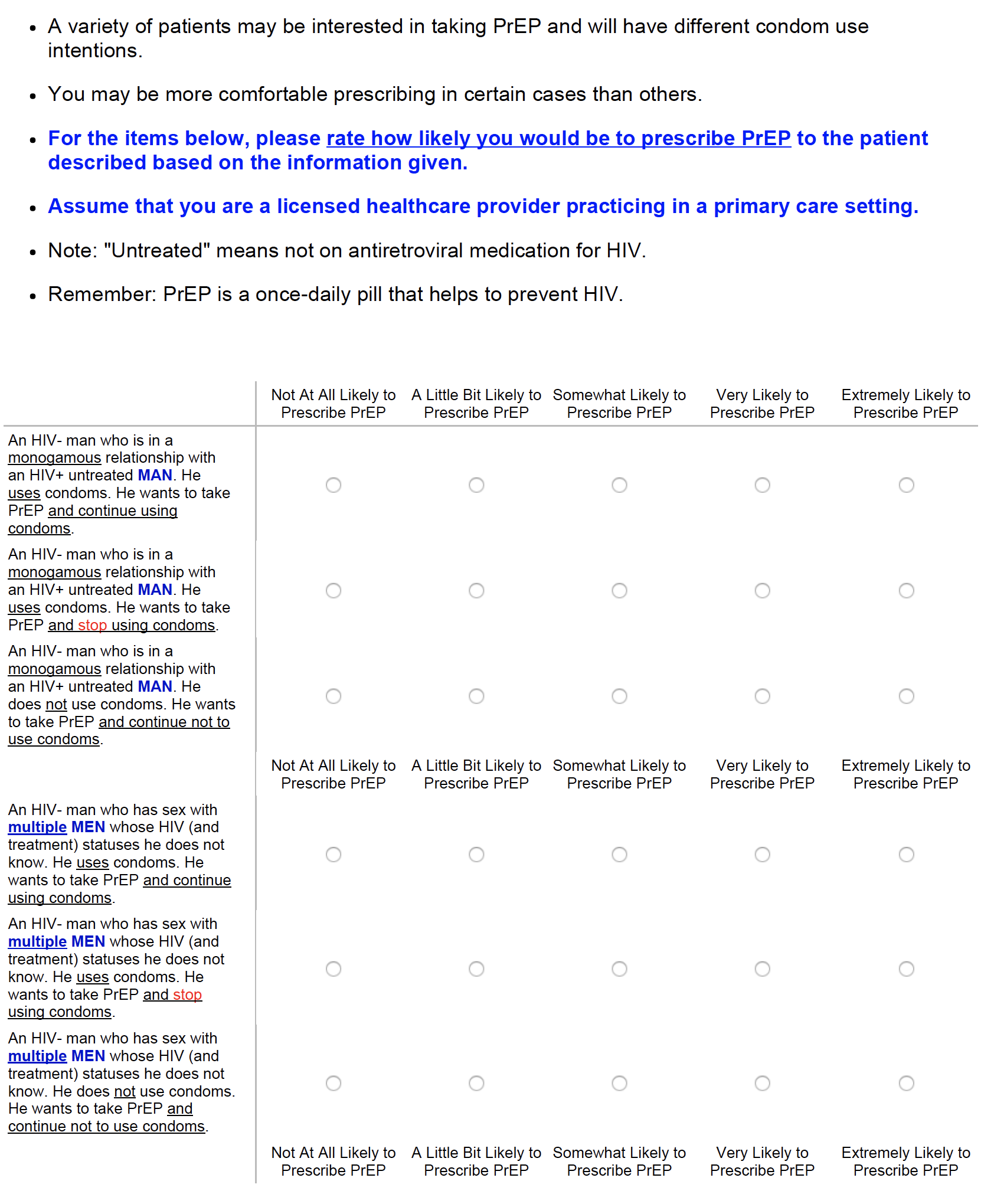


*[Perceived Acceptability of Reasons for Condom Discontinuation Measure]*


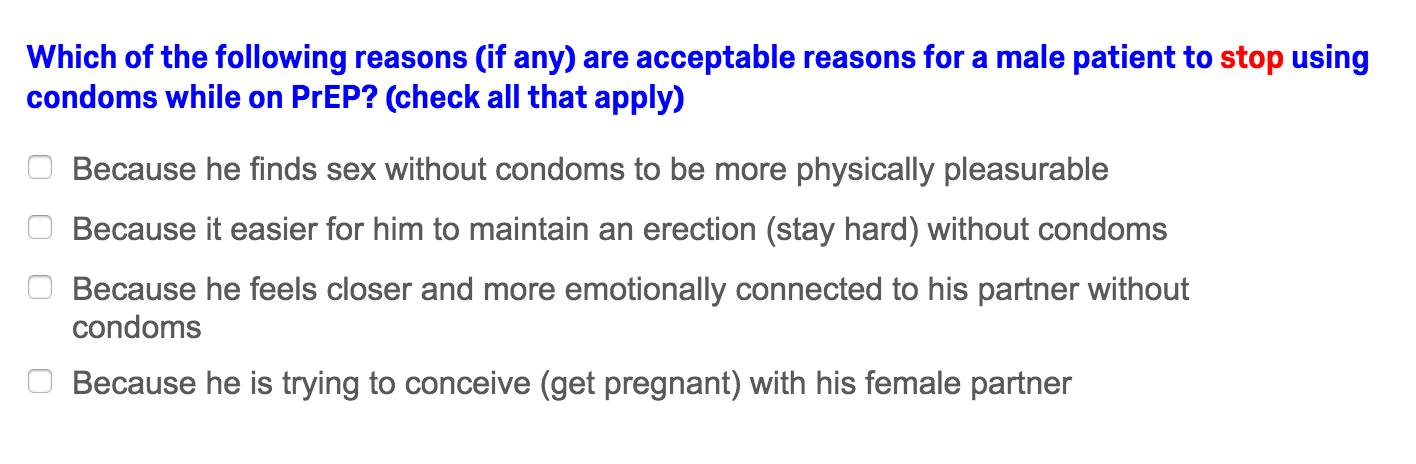


*[Debriefing Statement]*

****Please note that the supporting and opposing quotes related to PrEP that were presented in this study represent speculation and opinion. Scientific evidence from clinical trials suggests that, on average, people taking PrEP do NOT increase their risk behavior and that the risk of drug resistance is very low (0.1%) and not significantly different from placebo groups.**

*Note.* The debriefing statement above was included on the final page of the survey along with a link to the US Centers for Disease Control and Prevention website page for more information about PrEP ([link](http://www.cdc.gov/hiv/basics/prep.html)). Information about risk behavior and risk of drug resistance included in this statement was derived from the World Health Organization’s September 2015 guideline, *When to Start Antiretroviral Therapy and on Pre-Exposure Prophylaxis* ([link](http://apps.who.int/iris/bitstream/10665/186275/1/9789241509565_eng.pdf?ua=1)), which summarized PrEP trial evidence to date at the time the survey was developed.

**Online Supporting Information References**

1. Calabrese SK, Earnshaw VA, Underhill K, Hansen NB, Dovidio JF. The impact of patient race on clinical decisions related to prescribing HIV pre-exposure prophylaxis (PrEP): Assumptions about sexual risk compensation and implications for access. *AIDS Behav.* 2014;18:226-240.
2. Mayer KM, Ramjee G. The current status of the use of oral medication to prevent HIV transmission. *Curr Opin HIV AIDS*. 2015;10:226-232.
3. Krakower DS, Mayer KH. The role of healthcare providers in the roll out of preexposure prophylaxis. *Curr Opin HIV AIDS*. 2016;11:41-48.
4. Silapaswan A, Krakower D, Mayer KH. Pre-exposure prophylaxis: A narrative review of provider behavior and interventions to increase PrEP implementation in primary care. *J Gen Intern Med*. 2017;32:192-198.

1. Some of this supporting information was previously published in Calabrese SK, Earnshaw VA, Krakower DS, Underhill K, Vincent W, Magnus M, et al. A closer look at racism and heterosexism in medical students’ clinical decision-making related to HIV pre-exposure prophylaxis (PrEP): Implications for PrEP education. *AIDS Behav.* 2018;22:1122-38. [↑](#footnote-ref-1)
